# Two‐Nanosensor Electrochemical Profiling of Catecholamine Vesicle Interactions With Acute and Chronic Stress Granules in Living Cells

**DOI:** 10.1002/anie.202525900

**Published:** 2026-02-25

**Authors:** Hui Gu, Chaoyi Gu, Andre Du Toit, Andrew G. Ewing

**Affiliations:** ^1^ Department of Chemistry and Chemical Engineering Hunan University of Science and Technology Xiangtan 411201 China; ^2^ Department of Chemistry and Molecular Biology University of Gothenburg Gothenburg 41390 Sweden

**Keywords:** catecholamine vesicles, electrochemical nanosensors, neurodegeneration, reactive oxygen species (ROS), stress granules (SGs)

## Abstract

Stress granules (SGs) are dynamic, membrane‐less condensates that assemble in response to stress and have been increasingly linked to neurodegenerative disease (NDD) pathology. However, the molecular and functional mechanisms by which stress granules interact with other cellular organelles remain poorly understood. Here, we present a two‐nanosensor electrochemical strategy that enables quantitative discrimination of reactive oxygen species (ROS) in SGs and catecholamines in vesicles at single‐cell resolution. Using this approach, we distinguished the intracellular effects of acute and chronic SGs in catecholaminergic cells. Chronic SGs induced by prolonged cisplatin stress, contained elevated ROS levels and markedly increased catecholamine storage per vesicle, likely through ROS‐mediated homotypic vesicle fusion. In contrast, acute SGs induced by arsenite exhibited negligible effects. We further demonstrate that chronic SGs exhibit features of aged SGs, such as slow ROS release and enhanced redox activity. These results uncover a redox‐coupled SG‐vesicle interplay modulated by SG aging and identify chronic SGs as endogenous redox‐active condensates that may contribute to neurotransmitter dysregulation and neurodegeneration.

## Graphical abstract

A two‐nanosensor electrochemical strategy quantitatively distinguishes reactive oxygen species (ROS) in stress granules (SGs) from catecholamines in vesicles inside living cells. Chronic SGs formed under prolonged stress show elevated ROS and slower release, consistent with SG aging, and promote neurotransmitter loading likely through vesicle fusion, revealing a redox‐regulated link between SG aging, vesicle dysfunction, and neurodegeneration.

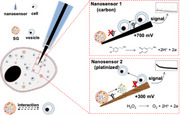

Stress granules (SGs) are critical membrane‐less organelles that assemble in response to diverse stressors, such as chemical agents (e.g., arsenite), ultraviolet radiation, and heat [1, 2]. These dynamic condensates are enriched in RNA and proteins through preferential sequestering from the cytoplasm, serving as adaptive compartments that enable cells to reorganize and regulate fundamental processes under stress [3, 4]. Increasing evidence implicates SGs in the pathogenesis of neurodegenerative diseases (NDDs), where they promote the aggregation of misfolded proteins, including transactive response (TAR) DNA‐binding protein 43 (TDP‐43) in amyotrophic lateral sclerosis (ALS) and frontotemporal dementia (FTD), and Tau in Alzheimer's disease (AD) [5, 6, 7, 8, 9, 10, 11, 12, 13].

Although SGs have been extensively characterized as protein‐RNA assemblies, much is still unknown about the contents of SGs, and how these impact function. We recently discovered that SGs possess intrinsic redox properties and can generate reactive oxygen species (ROS) through a spontaneous redox process mediated by the interfacial electric field at the SG‐cytoplasm interface, independent of enzymatic pathways [14, 15]. Furthermore, *ex‐vivo* experiments revealed that SGs can induce partial homotypic fusion of catecholamine‐containing vesicles, highlighting a redox‐driven mechanism of SG‐vesicle crosstalk relevant to NDD pathology [16].

These findings motivated us to probe SG‐vesicle interactions directly in living cells, where native granule properties and vesicle dynamics are preserved. However, this task presents a major analytical challenge: both ROS from SGs and catecholamines from synaptic vesicles are electroactive, often generating overlapping electrochemical signals. Thus, discriminating between these two species at the single‐cell level requires precise control of spatial resolution and redox resolution.

To overcome this, we leveraged the distinct physicochemical behaviors of SGs and vesicles. Catecholamines are confined within lipid‐bounded vesicles and require electroporation for release, whereas ROS in membrane‐less SGs are directly accessible to the nanosensor surface. Our previously established intracellular vesicle impact electrochemical cytometry (VIEC) technique exploits vesicle rupture upon impact to record quantal catecholamine release events [17, 18, 19]. In contrast, SGs yield spontaneous oxidation transients without electroporation, giving rise to a complementary single‐entity method termed stress granule impact electrochemical cytometry (SGIEC).

Building on this principle, we employed two electrochemical nanosensors with distinct redox properties and operating potentials to independently detect catecholamines in vesicles and ROS in SGs within living cells. Nanosensor 1, a carbon nanotip electrode, enabled selective detection of vesicular catecholamines (Figure S1). On its surface, dopamine was readily oxidized, whereas H_2_O_2_, the dominant ROS in SGs, remains electrochemically inert (Figure S2A,B). As expected, nanosensor 1 produced characteristic current spikes in vesicle suspensions but not in SGs at +700 mV vs. Ag/AgCl (Figure S3). To enable ROS detection, Nanosensor 2 was fabricated by platinizing nanosensor 1 (Figure S1), thereby promoting oxidation of both dopamine and H_2_O_2_ (Figure S2C,D). Notably, vesicle electroporation is strongly influenced by both surface properties and applied potential [20, 21]. Previous studies have shown that vesicles preferentially rupture on hydrophobic (e.g., thiolated gold) but remain intact on hydrophilic substrates (e.g., platinum) [22, 23, 24]. Therefore, we hypothesized that operating nanosensor 2 at a sufficiently low potential would allow selective detection of SG‐derived ROS while avoiding vesicle rupture.

We initially carried out electrochemical measurement of vesicles and SGs by nanosensor 2 at +400 mV (vs. Ag/AgCl) (a potential previously employed to detect SGs in our lab) [14, 15]. At this potential, current spikes were recorded continuously in both SG and vesicle suspension (Figure S4). However, it is worth noting that the frequency of the vesicle impact events was significantly reduced compared to those obtained at +700 mV with nanosensor 1 (Figure S2B). By further decreasing the potential to +350 mV and then +300 mV, the vesicle events obtained by VIEC were decreased and finally abolished at +300 mV (19 sensors were evaluated) (Table S1).

To validate selective SG detection, nanosensor 2 was sequentially immersed in vesicle and SG suspensions to perform VIEC and SGIEC. Only SGs yielded signal spikes, regardless of measurement order (Figure S5A,B), ruling out the possibility of delayed vesicle rupture on nanosensor 2. Moreover, when nanosensor 1 was operated at +300 mV for VIEC, the recorded spikes exhibited larger molecule counts and longer t_1/2_, suggesting that selective rupture of larger vesicles occurred due to greater membrane contact and electrostatic interaction (Figures S6 and S7) [25, 26, 27].

We next applied nanosensor 1 at +700 mV and nanosensor 2 at +300 mV (vs. Ag/AgCl) to a freshly mixed suspension of SGs and chromaffin vesicles for simultaneous detection. As shown in Figure 1A, amperometric traces at both sensors are characterized by a sequence of discrete current spikes, indicative of stochastic collision and oxidation events. Despite the presence of similar spike patterns, quantitative analysis revealed distinct signal profiles between the two nanosensors. Events detected by nanosensor 2 exhibited significantly lower molecular counts and shorter durations (t_1/2_) compared to those detected by nanosensor 1 (Figure 1B). These results are consistent with the patterns of VIEC and SGIEC performed separately by individual nanosensors in vesicular or SG suspensions (Figure S8A,B), confirming that the signals generated by nanosensor 1 and nanosensor 2 are mutually independent, and that no apparent crosstalk occurs between them under the mixed conditions. This independence is further supported by molecular number distribution analysis. As shown in Figure 1C, events detected by nanosensor 2 display a clear shift toward lower molecular numbers relative to vesicle‐derived events detected by nanosensor 1. Corresponding molecular number distributions obtained from separate vesicle‐only and SG‐only measurements are presented in Figure S8C,D and closely match those obtained under simultaneous detection conditions, further confirming that coexistence of vesicles and SGs does not distort the intrinsic electrochemical signatures of either entity. Our results establish a robust two‐nanosensor platform based on electrochemical signatures: nanosensor 1 at +700 mV enables selective detection of vesicle catecholamine content, while nanosensor 2 at +300 mV permits specific detection of SG‐derived ROS without vesicular interference. However, it is worth noting that the two‐nanosensor strategy supports simultaneous measurements in mixed suspensions, while spatial limitations necessitate independently optimized measurements in living cells.

**FIGURE 1:**
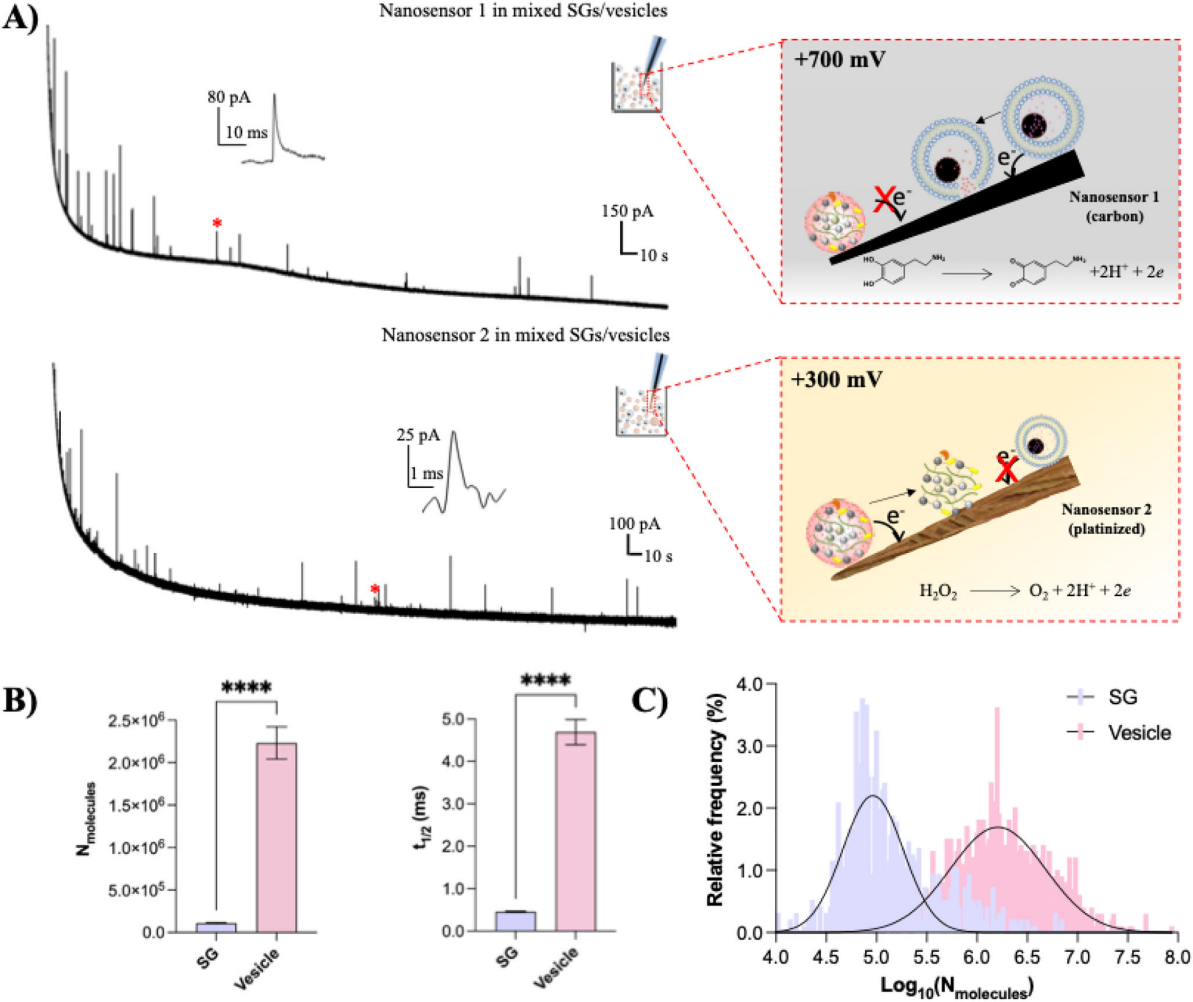
A) Representative vesicle impact electrochemical cytometry (VIEC) and stress granule impact electrochemical cytometry (SGIEC) amperometric traces recorded by nanosensor 1 at +700 mV (vs. Ag/AgCl, upper) and nanosensor 2 at +300 mV (vs. Ag/AgCl, lower) in a freshly mixed suspension of chromaffin vesicles and SGs. The inset highlights a representative spike (red asterisk). Right: schematic illustration of the selective quantification of vesicles and SGs with the two‐nanosensor system. B) Statistical analysis of the number of electroactive molecules (left) and t_1/2_ (right) obtained in panel A (n = 22 nanosensors per condition). C) Distribution of log_10_(N_molecules_) for SGs (n = 757 SGs) and vesicles (n = 896 vesicles), fitted using Gaussian models. Data are presented as mean ± SEM. Error bars represent SEM. Statistical significance was assessed using the Mann‐Whitney U test (two‐tailed) for unpaired, non‐normally distributed data: ****p < 0.0001.

Following the successful validation of our two‐nanosensor strategy in vitro, we next aimed to apply this platform for selective intracellular measurements of SGs and vesicles in living cells. Our initial attempt focused on chromaffin cells, a primary cell line derived from bovine adrenal medulla, known for their abundant secretory vesicles. We sought to induce SG formation in these cells using sodium arsenite, a well‐established oxidative stressor. However, even at elevated concentrations up to 1 mM, arsenite failed to trigger SG assembly in chromaffin cells, as confirmed by immunofluorescence imaging (Figure S9). This probably occurs because the high levels of antioxidant enzymes in chromaffin cells protect the cells from oxidative damage or because PLCβ1 might bind SG‐associated proteins and actively oppose SG assembly [28, 29, 30].

In PC12 cells, we successfully induced two distinct types of SGs by applying different stress regimens: 100 µM arsenite for 1 h to induce acute SGs, and 100 µM cisplatin for 24 h to induce chronic SGs (Figure 2). Arsenite‐induced SGs appeared rapidly (within 1 h) and disassembled within 1 h following stress removal, consistent with classical features of acute SGs [31, 32]. In contrast, cisplatin‐induced SGs persisted even at 12 h after stress removal, suggesting a more stable and stress‐resistant phenotype. These granules are classified as chronic SGs, which are typically formed under conditions of prolonged or pathological stress [33, 34]. Interestingly, cisplatin‐induced SGs appeared smaller than those induced by arsenite, suggesting chronic SGs are more solidified, probably due to stronger protein‐RNA crosslinking during maturation and aging during extended stress exposure [35, 36]. It is noteworthy that lower doses of cisplatin (e.g., 2 µM and 20 µM) were insufficient to induce visible SG formation in PC12 cells (Figure S10), further supporting the need for sustained, high‐level stress to generate chronic SG phenotypes suitable for intracellular analysis.

**FIGURE 2:**
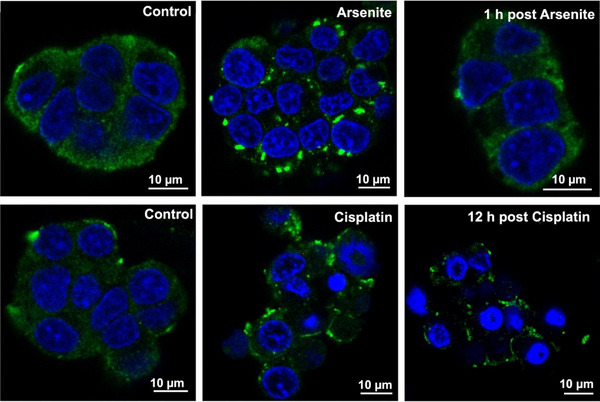
Representative fluorescence microscopy images of PC12 cells showing SG formation. Cells were treated with 100 µM arsenite for 1 h (acute SGs) or 100 µM cisplatin for 24 h (chronic SGs). Control cells were incubated with Dulbecco's phosphate‐buffered saline (DPBS) for 1 h or 24 h, respectively. For recovery experiments, treated cells were washed and incubated in fresh medium for 1 h (arsenite) or 12 h (cisplatin) prior to fixation. SGs were visualized by immunofluorescence staining of G3BP1 (green, a canonical SG marker).

To investigate how SGs influence vesicular behavior at the single‐cell level, we employed intracellular vesicle impact electrochemical cytometry (IVIEC) [17, 37, 38] using nanosensor 1. As shown in Figure 3A, the oxidation of catecholamines released from individual vesicles produced characteristic current spikes. It is worth noting that cells that had detached or exhibited obvious apoptotic morphology were excluded from electrochemical measurements. Quantitative analysis (Figure 3B) revealed that vesicles from PC12 cells containing chronic SGs exhibited significantly higher catecholamine content compared to untreated cells or those containing acute SGs. A similar trend was observed for t_1/2_, indicating a slower release dynamic possibly linked to increased vesicle size or fusion events. The log‐transformed molecular distributions fitted well to Gaussian models (Figure 3C), further confirmed an upward shift in vesicular content in the presence of chronic SGs. These findings support the hypothesis that chronic SGs may promote vesicle fusion, increasing transmitter storage capacity, a phenomenon we previously observed with *ex‐vivo* separated SGs [16]. Notably, low doses of cisplatin (2 µM or 20 µM) failed to increase vesicle content (Figure S11).

**FIGURE 3:**
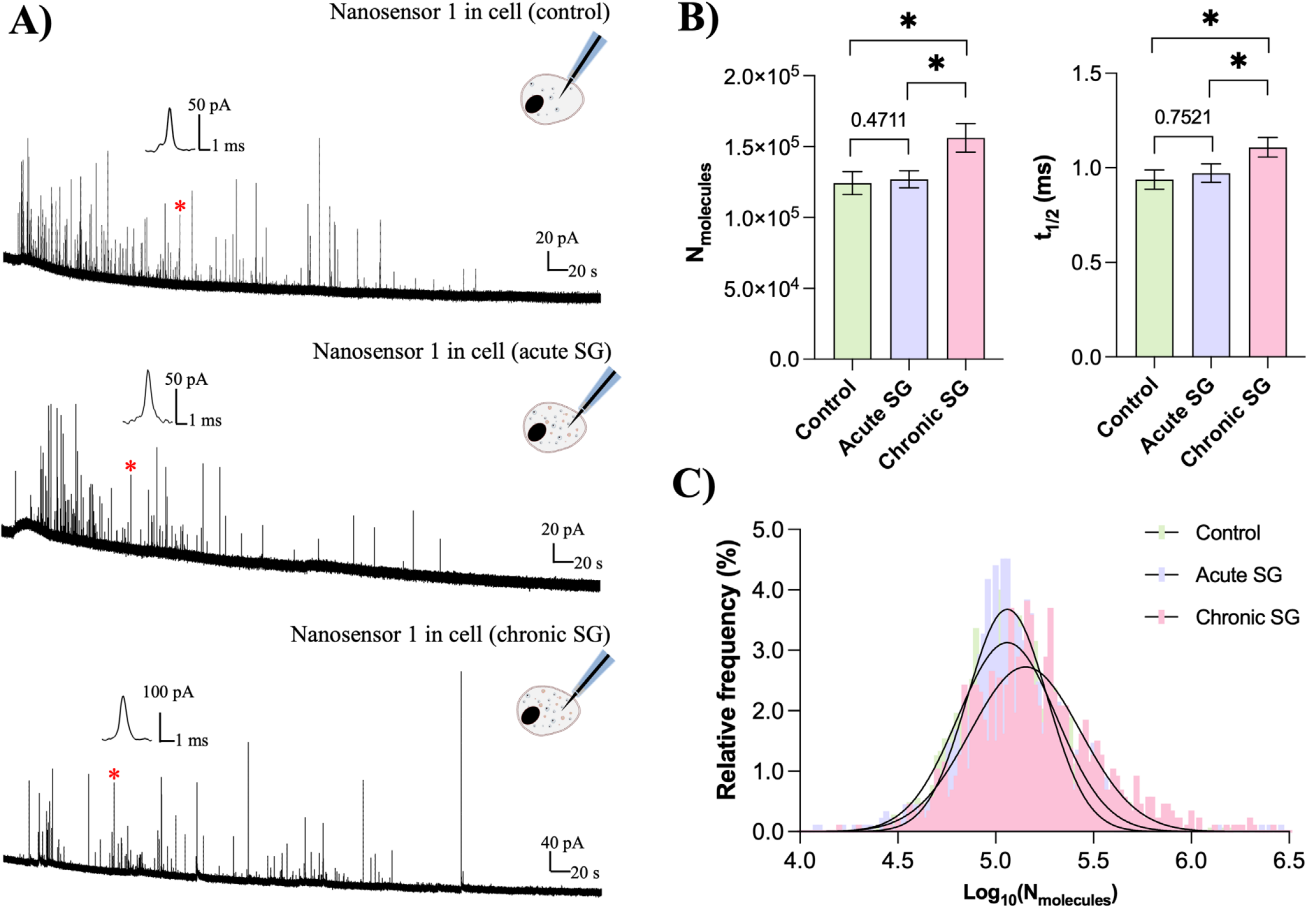
A) Representative intracellular vesicle impact electrochemical cytometry (IVIEC) amperometric traces obtained from nanosensor 1 at 700 mV vs. Ag/AgCl in PC12 cells before (upper) and after assembly of acute SGs (middle) and chronic SGs (lower). The inset shows an amplification of the spike labeled with the red asterisk. B) Bar graphs showing the statistical median for catecholamine molecules obtained in panel A (n = 17 cells for control, 16 cells for acute SGs, 10 cells for chronic SGs). C) Distributions of log_10_(N_molecules)_ for vesicles after the three treatments (n = 1042 vesicles for control, 936 vesicles for acute SGs, 573 vesicles for chronic SGs). Fits were obtained by fitting to a Gaussian distribution of the data. Data represent means ± SEM. Error bars represent SEM. Statistical significance was assessed using the Mann‐Whitney U test (two‐tailed) for unpaired, non‐normally distributed data: *, *p* < 0.05.

We next explored the redox profile of intracellular stress granules using intracellular SG impact electrochemical cytometry (ISGIEC) with nanosensor 2 operated at +300 mV. In untreated PC12 cells, background signals were minimal (Figure 4A, upper trace). Following SG assembly either acute SGs or chronic SGs, distinct current spikes were observed and attributed to the oxidation of reactive oxygen species (ROS) released from SGs (Figure 4A, lower traces). The frequency of ROS‐related spikes was lower than that observed for vesicles, likely reflecting the lower abundance of SGs or reduced interaction probability with the nanosensor at this operating potential. As shown in Figure 4B,C, acute SGs exhibit relatively low ROS signals with fast decay kinetics, whereas chronic SGs display significantly higher ROS content accompanied by markedly slower release dynamics. These features indicate enhanced interfacial electrochemical activity of chronic SGs compared to acute SGs, consistent with our previous findings [15]. The substantially slower ROS release from chronic SGs further suggests a more solidified and structurally stabilized condensate state, which restricts ROS transport toward the nanosensor interface and is characteristic of SG aging.

**FIGURE 4:**
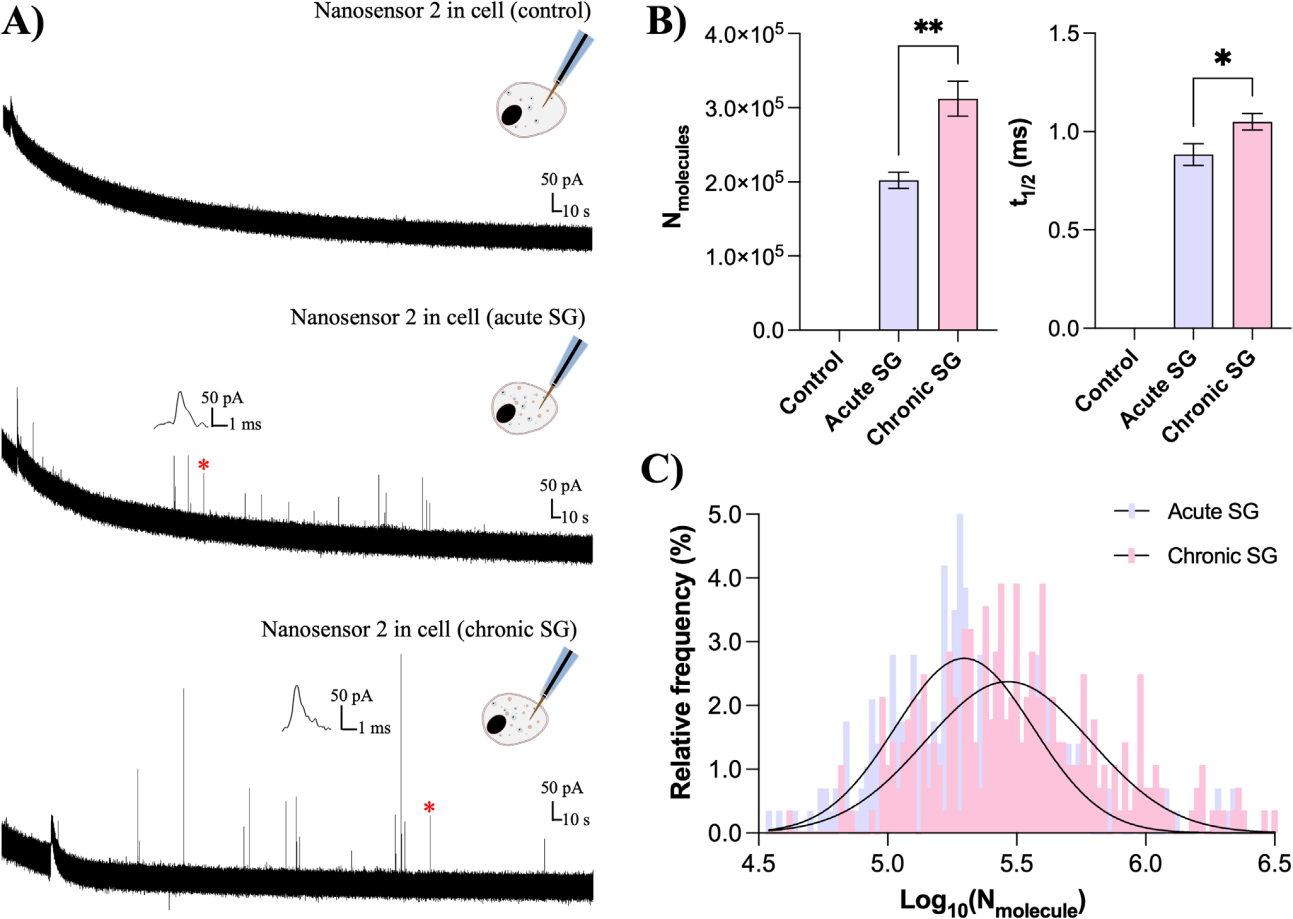
A) Representative intracellular SG impact electrochemical cytometry (ISGIEC)amperometric traces obtained from electrochemical nanosensor 2 at +300 mV vs. Ag/AgCl in PC12 cells before (upper) and after assembly of acute SGs (middle) and chronic SGs (lower). The inset shows an amplification of the spike labeled with the red asterisk. B) Bar graphs show the statistical median for ROS molecules obtained in panel A (n = 10 cells for control, 15 cells for acute SGs, 14 cells for chronic SGs). C) Distribution of log_10_(N_molecules_) for SGs (n = 4 spikes for control, 300 SGs for acute SGs, 294 SGs for chronic SGs). Fits were obtained assuming a Gaussian distribution of the data. Data represent means ± SEM. Error bars represent SEM. Statistical significance was assessed using the Mann‐Whitney U test (two‐tailed) for unpaired, non‐normally distributed data:*, *p* < 0.05; **, *p* < 0.01.

Previous studies have shown that protein‐rich condensates with complex and heterogeneous compositions experience substantial reorganization of their internal architectures during maturation and aging, which leads to the gradual loss of their liquid‐like characteristics and disequilibrium of electrochemical dynamics over time [39, 40, 41, 42]. Motivated by this, we hypothesized that the elevated ROS content and slower release kinetics of chronic SGs may arise from similar aging‐associated structural transitions. To test this, we isolated typical acute SGs from U2OS cells and subjected them to aging at 4 °C for 72 h to slow ROS degradation. Using nanosensor 2, we then performed SGIEC to determine the change in thecontained ROS levels over time. As displayed in Figure S12A, ROS content initially decreased during the first 24 h likely due to ROS degradation or SG disassembly but increased markedly between 48 h and 72 h, indicating that ROS generation at SGs is enhanced during aging. Meanwhile, the release kinetics of ROS became progressively slower (Figure S12B), suggesting that SGs gradually undergo solidification as they age. This transition likely results from enhanced crosslinking between internal RNAs and proteins, which promotes structural condensation and stabilization. Notably, chronic SGs induced by cisplatin display these same hallmarks, implying that they represent an aged or matured state of SGs.

Mechanistically, sustained ROS exposure during aging can oxidize amino acid residues and promote covalent protein crosslinking [43, 44], disrupting the equilibrium of interfacial electrochemistry and consequently promoting additional ROS generation [42, 45, 46, 47, 48]. This feedback loop establishes a self‐reinforcing process in which ROS‐driven crosslinking and structural condensation enhance the redox activity and solidification of SGs. The resulting ROS‐enriched microenvironment enhances local oxidative imbalance, facilitating the irreversible transition from liquid‐like acute SGs to solid‐like chronic SGs.

Pathologically, this mechanism offers a compelling explanation for the functional divergence between acute and chronic SGs. While acute SGs exert minimal effects on vesicular neurotransmitter storage, chronic SGs markedly increase catecholamine content, likely through homotypic vesicle fusion [15]. Given that vesicular dysfunction is a convergent mechanism in numerous neurodegenerative diseases (NDDs) [49, 50], our results suggest that chronic SGs may actively contribute to neurotransmitter dysregulation during disease progression. Moreover, the ROS‐enriched microenvironment can directly promote protein misfolding and aggregation [51, 52], providing a mechanistic bridge between SG aging and neurodegenerative pathology. In line with this, disulfide bond formation within TDP‐43 proteins in SGs has been shown to trigger local aggregation, a molecular hallmark of amyotrophic lateral sclerosis (ALS) and frontotemporal dementia (FTD) in a redox‐dependent manner [53]. Collectively, our findings establish chronic SGs as endogenous redox‐active condensates that perpetuate oxidative stress and structural aging, offering new insight into how persistent SGs might act as active participants in the molecular pathogenesis of neurodegenerative diseases.

In summary, our work establishes a two‐nanosensor electrochemical strategy for quantitatively discriminating ROS‐enriched SGs and catecholamine‐containing vesicles within living cells. This dual‐entity measurement reveals that chronic SGs possess stronger redox activity and slower ROS release kinetics than acute SGs, consistent with their aged and structurally solidified state. Functionally, these chronic SGs promote catecholamine accumulation in vesicles, likely by inducing ROS‐driven vesicle fusion. Together, these findings elucidate how SG aging reshapes intracellular redox microenvironments and vesicle dynamics, suggesting a mechanistic pathway through which persistent SGs may contribute to neurotransmission imbalance and the molecular pathogenesis of neurodegenerative diseases. Beyond uncovering this biological interplay, our work establishes a versatile electrochemical platform for probing redox‐regulated organelle interactions in living cells.

## Conflicts of Interest

The authors declare no conflicts of interest.

## Supporting information




**Supporting File 1**: The authors have cited additional references within the Supporting Information [14–18, 54].

## Data Availability

The data that support the findings of this study are available from the corresponding author upon reasonable request.

## References

[1] D. S. W. Protter and R. Parker , “Principles and Properties of Stress Granules,” Trends in Cell Biology 26 (2016): 668–679, 10.1016/j.tcb.2016.05.004. 27289443 PMC4993645

[2] T. Hirose , K. Ninomiya , S. Nakagawa , and T. Yamazaki , “A Guide to Membraneless Organelles and Their Various Roles in Gene Regulation,” Nature Reviews Molecular Cell Biology 24 (2023): 288–304, 10.1038/s41580-022-00558-8. 36424481

[3] P. Ivanov , N. Kedersha , and P. Anderson , “Stress Granules and Processing Bodies in Translational Control,” Cold Spring Harbor Perspectives in Biology 11 (2019): a032813, https://cshperspectives.cshlp.org/content/11/5/a032813.long. 30082464 10.1101/cshperspect.a032813 PMC6496347

[4] H. Yoo , C. Triandafillou , and D. A. Drummond , “Cellular Sensing by Phase Separation: Using the Process, Not Just the Products,” Journal of Biological Chemistry 294 (2019): 7151–7159, 10.1074/jbc.TM118.001191. 30877200 PMC6509497

[5] Q. Cui , Z. Liu , and G. Bai , “Friend or Foe: The Role of Stress Granule in Neurodegenerative Disease,” Neuron 112 (2024): 2464–2485, 10.1016/j.neuron.2024.04.025. 38744273

[6] Y. Khalfallah , R. Kuta , C. Grasmuck , A. Prat , H. D. Durham , and C. Vande Velde , “TDP‐43 Regulation of Stress Granule Dynamics in Neurodegenerative Disease‐Relevant Cell Types,” Scientific Reports 8 (2018): 7551. 29765078 10.1038/s41598-018-25767-0 PMC5953947

[7] Z. Wang , C. Yang , X. Wang , et al., “Decoding Stress Granules Dynamics: Implications for Neurodegenerative Disease,” Progress in Neurobiology 248 (2025): 102758, 10.1016/j.pneurobio.2025.102758. 40132681

[8] L. Yuan , L.‐H. Mao , Y.‐Y. Huang , et al., “Stress Granules: Emerging Players in Neurodegenerative Diseases,” Translational Neurodegeneration 14 (2025): 22. 40355949 10.1186/s40035-025-00482-9 PMC12067921

[9] Y. R. Li , O. D. King , J. Shorter , and A. D. Gitler , “Stress Granules as Crucibles of ALS Pathogenesis,” Journal of Cell Biology 201 (2013): 361–372, 10.1083/jcb.201302044. 23629963 PMC3639398

[10] M. Ramaswami , J. P. Taylor , and R. Parker , “Altered Ribostasis: RNA‐Protein Granules in Degenerative Disorders,” Cell 154 (2013): 727–736, 10.1016/j.cell.2013.07.038. 23953108 PMC3811119

[11] V. Tara , Y. Haung , V. Megan , et al., “Contrasting Pathology of the Stress Granule Proteins TIA‐1 and G3BP in Tauopathies,” Journal of Neuroscience 32 (2012): 8270. 22699908 10.1523/JNEUROSCI.1592-12.2012 PMC3402380

[12] M. R. Asadi , M. Sadat Moslehian , H. Sabaie , et al., “Stress Granules and Neurodegenerative Disorders: A Scoping Review,” Frontiers in Aging Neuroscience 13 (2021): 650740. 34248597 10.3389/fnagi.2021.650740 PMC8261063

[13] C. B. Trengrove 2016, “Autophagy and Stress Granules: The Merging of Two Pathways in Parkinson's Disease.” PhD diss., Boston University.

[14] K. Hu , E. Relton , N. Locker , N. T. N. Phan , and A. G. Ewing , “Electrochemical Measurements Reveal Reactive Oxygen Species in Stress Granules**,” Angewandte Chemie International Edition 60 (2021): 15302–15306, 10.1002/anie.202104308. 33876544 PMC8456511

[15] H. Gu , C. Gu , A. Du Toit , et al., “Single‐Entity Resolution Single‐Cell Nanosensor Reveals Reactive Oxygen Species at Stress Granules Are Formed by Interfacial Redox Chemistry,” Journal of the American Chemical Society 147 (2025): 27020–27029, 10.1021/jacs.5c09338. 40692136 PMC12314916

[16] H. Gu , C. Gu , N. Locker , and A. G. Ewing , “Amperometry and Electron Microscopy Show Stress Granules Induce Homotypic Fusion of Catecholamine Vesicles,” Angewandte Chemie International Edition 63 (2024): e202400422, 10.1002/anie.202400422. 38380500

[17] X. Li , S. Majdi , J. Dunevall , H. Fathali , and A. G. Ewing , “Quantitative Measurement of Transmitters in Individual Vesicles in the Cytoplasm of Single Cells With Nanotip Electrodes,” Angewandte Chemie International Edition 54 (2015): 11978–11982, 10.1002/anie.201504839. 26266819 PMC4747609

[18] J. Dunevall , H. Fathali , N. Najafinobar , et al., “Characterizing the Catecholamine Content of Single Mammalian Vesicles by Collision–Adsorption Events at an Electrode,” Journal of the American Chemical Society 137 (2015): 4344–4346, 10.1021/ja512972f. 25811247

[19] X. Li , L. Ren , J. Dunevall , et al., “Nanopore Opening at Flat and Nanotip Conical Electrodes During Vesicle Impact Electrochemical Cytometry,” ACS Nano 12 (2018): 3010–3019, 10.1021/acsnano.8b00781. 29513514

[20] X. Li , J. Dunevall , L. Ren , and A. G. Ewing , “Mechanistic Aspects of Vesicle Opening During Analysis With Vesicle Impact Electrochemical Cytometry,” Analytical Chemistry 89 (2017): 9416–9423, 10.1021/acs.analchem.7b02226. 28776974

[21] J. Lovrić , N. Najafinobar , J. Dunevall , et al., “On the Mechanism of Electrochemical Vesicle Cytometry: Chromaffin Cell Vesicles and Liposomes,” Faraday Discussions 193 (2016): 65–79. 27711871 10.1039/c6fd00102e

[22] E. Lebègue , C. M. Anderson , J. E. Dick , L. J. Webb , and A. J. Bard , “Electrochemical Detection of Single Phospholipid Vesicle Collisions at a Pt Ultramicroelectrode,” Langmuir 31 (2015): 11734–11739. 26474107 10.1021/acs.langmuir.5b03123

[23] K. Dimitrievski and B. Kasemo , “Simulations of Lipid Vesicle Adsorption for Different Lipid Mixtures,” Langmuir 24 (2008): 4077–4091, 10.1021/la703021u. 18318551

[24] C. A. Keller and B. Kasemo , “Surface Specific Kinetics of Lipid Vesicle Adsorption Measured With a Quartz Crystal Microbalance,” Biophysical Journal 75 (1998): 1397–1402, 10.1016/S0006-3495(98)74057-3. 9726940 PMC1299813

[25] T. L. Colliver , S. J. Pyott , M. Achalabun , and G. E. Andrew , “VMAT‐Mediated Changes in Quantal Size and Vesicular Volume,” Journal of Neuroscience 20 (2000): 5276–5282, 10.1523/JNEUROSCI.20-14-05276.2000. 10884311 PMC6772308

[26] H. Fathali , J. Dunevall , S. Majdi , and A.‐S. Cans , “Extracellular Osmotic Stress Reduces the Vesicle Size While Keeping a Constant Neurotransmitter Concentration,” ACS Chemical Neuroscience 8 (2017): 368–375, 10.1021/acschemneuro.6b00350. 27966899

[27] X. Zhang and A. G. Ewing , “Pore‐Opening Dynamics of Single Nanometer Biovesicles at an Electrified Interface,” American Chemical Society Nano 16 (2022): 9852–9858, 10.1021/acsnano.2c03929. 35647887 PMC9245343

[28] A. Qifti , L. Jackson , A. Singla , O. Garwain , and S. Scarlata , “Stimulation of Phospholipase Cβ1 by Gαq Promotes the Assembly of Stress Granule Proteins,” Science Signaling 14 (2021): eaav1012, 10.1126/scisignal.aav1012. 34665639

[29] M. Basu , S. C. Courtney , and M. A. Brinton , “Arsenite‐Induced Stress Granule Formation Is Inhibited by Elevated Levels of Reduced Glutathione in West Nile Virus‐Infected Cells,” Public Library of Science Pathogens 13 (2017): e1006240, 10.1371/journal.ppat.1006240. PMC5344523 28241074

[30] A.‐B. Blázquez , M. A. Martín‐Acebes , T. Poderoso , and J.‐C. Saiz , “Relevance of Oxidative Stress in Inhibition of eIF2 Alpha Phosphorylation and Stress Granules Formation During Usutu Virus Infection,” PLOS Neglected Tropical Diseases 15 (2021): e0009072, 10.1371/journal.pntd.0009072. 33493202 PMC7861526

[31] L. C. Reineke and J. R. Neilson , “Differences Between Acute and Chronic Stress Granules, and How these Differences May Impact Function in Human Disease,” Biochemical Pharmacology 162 (2019): 123–131, 10.1016/j.bcp.2018.10.009. 30326201 PMC6421087

[32] A. Aulas , M. M. Fay , S. M. Lyons , et al., “Stress‐Specific Differences in Assembly and Composition of Stress Granules and Related Foci,” Journal of Cell Science 130 (2017): 927–937, 10.1242/jcs.199240. 28096475 PMC5358336

[33] A. Ratti , V. Gumina , P. Lenzi , et al., “Chronic Stress Induces Formation of Stress Granules and Pathological TDP‐43 Aggregates in Human ALS Fibroblasts and IPSC‐Motoneurons,” Neurobiology of Disease 145 (2020): 105051, 10.1016/j.nbd.2020.105051. 32827688

[34] Y. Adachi , A. M. Williams , M. Masuda , Y. Taketani , P. J. Anderson , and P. Ivanov , “Chronic Stress Antagonizes Formation of Stress Granules,” iScience 29 (2026): 114556. 41583565 10.1016/j.isci.2025.114556 PMC12828549

[35] M. Linsenmeier , M. Hondele , F. Grigolato , E. Secchi , K. Weis , and P. Arosio , “Dynamic Arrest and Aging of Biomolecular Condensates Are Modulated by Low‐Complexity Domains, RNA and Biochemical Activity,” Nature Communications 13 (2022): 3030. 10.1038/s41467-022-30521-2 PMC9156751 35641495

[36] W. Yu , X. Guo , Y. Xia , et al., “Aging‐Dependent Evolving Electrochemical Potentials of Biomolecular Condensates Regulate Their Physicochemical Activities,” Nature Chemistry 17 (2025): 756–766, 10.1038/s41557-025-01762-7. 40074825

[37] C. Gu , Y. Wang , M. Yeoman , B. A. Patel , and A. G. Ewing , “Subsets of Nanometer Vesicles in the Fly Release Differential Fractions of Vesicular Serotonin Content During Exocytosis,” Angewandte Chemie International Edition 63 (2024): e202409783, 10.1002/anie.202409783. 39101881

[38] Y. Wang , C. Gu , and A. G. Ewing , “Single‐Vesicle Electrochemistry Following Repetitive Stimulation Reveals a Mechanism for Plasticity Changes With Iron Deficiency,” Angewandte Chemie International Edition 61 (2022): e202200716, 10.1002/anie.202200716. 35267233 PMC9315038

[39] A. Patel , H. O. Lee , L. Jawerth , et al., “A Liquid‐to‐Solid Phase Transition of the ALS Protein FUS Accelerated by Disease Mutation,” Cell 162 (2015): 1066–1077. 26317470 10.1016/j.cell.2015.07.047

[40] S. Alberti and D. Dormann , “Liquid–Liquid Phase Separation in Disease,” Annual Review of Genetics 53 (2019): 171–194, 10.1146/annurev-genet-112618-043527. 31430179

[41] A. Garaizar , J. R. Espinosa , J. A. Joseph , et al., “Aging Can Transform Single‐Component Protein Condensates Into Multiphase Architectures,” PNAS 119 (2022): e2119800119, 10.1073/pnas.2119800119. 35727989 PMC9245653

[42] W. Yu , X. Guo , Y. Xia , et al., “Aging‐Dependent Evolving Electrochemical Potentials of Biomolecular Condensates Regulate Their Physicochemical Activities,” Nature Chemistry 17 (2025): 756–766, 10.1038/s41557-025-01762-7. 40074825

[43] H. Wang , B. Favetta , B. S. Schuster , and Z. Shi , “BPS2025 – Reactive Oxygen Species Solidify Protein Condensates,” Biophysical Journal 124 (2025): 75a.

[44] M. W. Chen , X. Ren , X. Song , et al., “Transition‐State‐Dependent Spontaneous Generation of Reactive Oxygen Species by Aβ Assemblies Encodes a Self‐Regulated Positive Feedback Loop for Aggregate Formation,” Journal of the American Chemical Society 147 (2025): 8267–8279, 10.1021/jacs.4c15532. 39999421

[45] Y. Dai , Z. Zhou , W. Yu , et al., “Biomolecular Condensates Regulate Cellular Electrochemical Equilibria,” Cell 187 (2024): 5951–5966, e5918. 39260373 10.1016/j.cell.2024.08.018 PMC11490381

[46] D. D. Lee , L. Galera‐Laporta , M. Bialecka‐Fornal , et al., “Magnesium Flux Modulates Ribosomes to Increase Bacterial Survival,” Cell 177 (2019): 352–360, e313. 30853217 10.1016/j.cell.2019.01.042 PMC6814349

[47] K. Kikuchi , L. Galera‐Laporta , C. Weatherwax , et al., “Electrochemical Potential Enables Dormant Spores to Integrate Environmental Signals,” Science 378 (2022): 43–49. 36201591 10.1126/science.abl7484 PMC10593254

[48] J. Humphries , L. Xiong , J. Liu , et al., “Species‐Independent Attraction to Biofilms Through Electrical Signaling,” Cell 168 (2017): 200–209, e212. 28086091 10.1016/j.cell.2016.12.014 PMC5497501

[49] P. A. Lewis , “Vesicular Dysfunction and Pathways to Neurodegeneration,” Essays in Biochemistry 65 (2021): 941–948. 10.1042/EBC20210034 PMC8709888 34897416

[50] T. Logan , J. Bendor , C. Toupin , K. Thorn , and R. H. Edwards , “α‐Synuclein Promotes Dilation of the Exocytotic Fusion Pore,” Nature Neuroscience 20 (2017): 681–689, 10.1038/nn.4529. 28288128 PMC5404982

[51] D. M. Teleanu , A. G. Niculescu , Lungu, II , et al., “An Overview of Oxidative Stress, Neuroinflammation, and Neurodegenerative Diseases,” International Journal of Molecular Sciences 23 (2022): 5938, 10.3390/ijms23115938. 35682615 PMC9180653

[52] A. Houldsworth , “Role of Oxidative Stress in Neurodegenerative Disorders: A Review of Reactive Oxygen Species and Prevention by Antioxidants,” Brain Communications 6 (2024): fcad356. 38214013 10.1093/braincomms/fcad356 PMC10783645

[53] X. Yan , D. Kuster , P. Mohanty , et al., “Intra‐Condensate Demixing of TDP‐43 Inside Stress Granules Generates Pathological Aggregates,” Cell 188 (2025): 4123–4140.e18. 40412392 10.1016/j.cell.2025.04.039 PMC12303766

