# Supplementary material for: Two‐Nanosensor Electrochemical Profiling of Catecholamine Vesicle Interactions With Acute and Chronic Stress Granules in Living Cells
Source: Angew Chem Int Ed Engl. 2026 Feb 25;65(15):e25900. doi: 10.1002/anie.202525900 (PMC13053923; doi:10.1002/anie.202525900)
Supplement: Supplementary file 1 — Supporting File 1: The authors have cited additional references within the Supporting Information [14–18, 54]. [file ANIE-65-e25900-s001.pdf]

Two-Nanosensor Electrochemical Profiling of Catecholamine Vesicle Interactions with Acute and Chronic Stress Granules in Living Cells

Hui Gu,[a,b]*, Chaoyi Gu,[b] Andre Du Toit,[b] Andrew G Ewing[b]*

[a] Prof. Hui Gu
Department of Chemistry and Chemical Engineering, Hunan University of Science and Technology, Xiangtan, 411201, China.
E-mail: hgu@hnust.edu.cn

[b] Prof. Hui Gu, Dr. Chaoyi Gu, Dr. Andre Du Toit, Prof. Andrew G Ewing
Department of Chemistry and Chemical Engineering, Hunan University of Science and Technology, Xiangtan, 411201, China.
E-mail: andrewe@chem.gu.se

List of Contents

1. **Experimental Section**
2. **Supporting Figures and Tables**

Figure S1. Schematic illustration of nanosensor fabrication.

Figure S2. Cyclic voltammograms of nanosensors in PBS with dopamine and H_2O_2.

Figure S3. Amperometric traces of nanosensor 1 at +700 mV in SG and vesicle suspensions.

Figure S4. Amperometric traces of nanosensor 2 at +400 mV in SG and vesicle suspensions.

Table S1. Statistical summary of spike counts at various applied potentials.

Figure S5. Sequential detection of vesicle and SG signals by nanosensor 2.

Figure S6–S7. Vesicle parameters comparison at +300 mV and +700 mV.

Figure S8. Comparison of vesicle and SG parameters detected separately.

Figure S9. Immunofluorescence images of chromaffin cells treated with arsenite.

Figure S10. Immunofluorescence images of PC12 cells treated with cisplatin.

Figure S11. Catecholamine content per vesicle under different cisplatin concentrations.

Figure S12. ROS evolution and release dynamics in aged SGs stored at 4 °C.

3. **References**

1. Experimental section

1.1. Chemicals

All chemicals were analytical grade, purchased from Sigma-Aldrich and used as received. 18 MΩ·cm water from Purelab Classic purification (ELGA, Sweden) was used to prepare all solutions. 1× Locke's buffer was prepared by diluting 10× Locke's buffer 1540 mM NaCl, 56 mM KCl, 36 mM $NaHCO_3$, 56 mM glucose, 50 mM HEPES, 1% (v/v) penicillin before use. SG lysis buffer included 50 mM Tris-HCl, 100 mM potassium acetate, 2 mM magnesium acetate, 0.5 mM DTT, 50 µg/mL heparin, 0.5 % NP-40, 1 complete mini EDTA-free protease inhibitor tablet. Homogenizing buffer (pH 7.4) contained 0.25 M sucrose, 1 mM EDTA, 1 mM $MgSO_4$, 10 mM HEPES, 10 mM KCl. The isotonic saline solution (pH 7.4) consisted of 150 mM NaCl, 5 mM KCl, 1.2 mM $MgCl_2$, 2 mM $CaCl_2$, 5 mM glucose and 10 mM HEPES. pH of all solutions was adjusted to 7.4 with 3M NaOH and solutions were filtered before use.

1.2. Cell culture

U2OS cell culture: High glucose Dulbecco's modified Eagle's medium (DMEM) supplemented with 10% fetal bovine serum (FBS), 1 % penicillin-streptomycin and 1ug/mL puromycin was used to maintain Human bone osteosarcoma epithelial (U2OS) cells. The cells were grown on TC-treated T75 flasks (Sarstedt, Sweden) and cultured at 37°C in a 5 % CO_2, 100% humidity incubator.

PC12 cell culture: RPMI-1640 medium supplemented with 10% donor horse serum and 5% fetal bovine serum was used to maintain PC12 cells (gift from Lloyd Greene). The cells were grown on T25 flasks coated with 0.1 mg/mL human placenta collagen and for the electrochemistry experiment, cells were seeded on 60 mm dishes with the same type of coating. The cells were cultured at 37 °C in a 5% CO_2 and 100% humidity incubator.

1.3. Isolation of adrenal chromaffin vesicles

Chromaffin vesicles were isolated according to the previously reported protocol.[17-18] First, fresh bovine adrenal glands were obtained from a local slaughterhouse and were cleaned with 1× Locke's buffer to remove blood. Then, the glands were cut into two and the medulla parts were removed from the gland and transferred to a homogenizing buffer, which was followed by mechanical homogenization with a homogenizer (Wheaton, U.S.A.). Finally, a series of centrifugation steps at 4 °C was used to purify the vesicles: 1) 1000 g for 10 min to remove extra blood cells; 2) 10000 g for 20 min to pellet vesicles. The obtained pellet of vesicles was resuspended in the homogenizing buffer for further use on the same day.

1.4. Isolation of SGs

The SGs were isolated according to previously reported protocol.[14, 16] First, U2OS cells were stressed with 0.1 mM sodium arsenite for 1 h and spun at 230 g for 5 min. Then, the obtained pellet was resuspended in 1 ml of SG lysis buffer and snap frozen. The suspension was lysed by passing through a 25 G 5/8 needle seven times on ice and then spun at 1,000 g for 5 min. The supernatant containing SGs was centrifuged at 18,000 g for 20 min to obtain the SGs pellet. This pellet was resuspended in 1 mL of All the centrifugation steps were performed at 4 °C. Finally, the purified SGs were resuspended in 100 µL of homogenizing buffer for further use.

1.5. Immunofluorescence microscopy imaging of PC12 cells

PC12 cells were grown on glass bottom dishes (MatTek), followed by 100 µm arsenite for 1 h or 100 µm cisplatin for 24 h. To check the disassembly of SGs, arsenite or cisplatin was removed, and the cells were maintained in medium for 1 to 12 h. After the cells were ready, they were washed a few times with PBS. Then the cells were fixed by keeping the cells in a solution of 4 % PFA in PBS (Fisher Scientific, Sweden) for 20 min at room temperature (RT), and stored at 4°C. The cells were subsequently washed with 100 mM glycine for 20 min and with PBS 3 times at RT. Afterwards, the cells were permeabilized and blocked with a mixture of 2 % bovine serum albumin (BSA) and 0.1 % Triton X-100 in PBS (BSA/Tri/PBS) for 60 min at RT. Then, the cells were incubated with monoclonal mouse anti-human in BSA/Tri/PBS (ratio 1:200) solution for 60 min and washed three times with BSA/Tri/PBS solution for 5 min at RT. Incubated samples with a secondary antibody solution consisting of Abberior STAR 635 anti-mouse goat antibody (Abberior) in BSA/Tri/PBS (ratio 1:500) for 60 min at RT, followed by washing with PBS containing DAPI (ThermoFisher) (ratio 1:1000) for 5 min and rinsing for another 5 min. Confocal microscopy was performed on a Ti-Eclipse-A1MP Multiphoton Confocal Microscope (Nikon) using the Nikon acquisition software NIS-Elements AR. PC12 cells without arsenite treatment serve as the control group and PC12 cells with 1 h arsenite treatment, but lacking incubation with monoclonal mouse anti-human antibody, served as the negative group to conduct imaging for comparison.

1.7. Fabrication of electrochemical nanosensors

Preparation of nanosensor 1: Fabrication of nanosensor 1 was previously described.[1, 2] First, a 5-µm diameter carbon fiber was aspirated into a borosilicate capillary (1.2 mm O.D., 0.69 mm I.D., Sutter Instrument Co., Novato, CA, USA). Then, the capillary was pulled in half with a micropipette puller (model PE-21, Narishige, Inc., Japan) to obtain two electrodes. Next, the fiber extending from the glass was cut to 100-150 µm with a scalpel under a microscope. The electrodes were subsequently held on the edge of the blue part of a butane flame (Clas Ohlson, Sweden) to flame etch until a needle-sharp tip with about 50-200 nm tip diameter was obtained. The electrodes were sealed by dipping the tip into epoxy solution (GA Lindberg ChemTech AB, Sweden), followed by drying in an oven at 100°C overnight. Before use, good electrodes were sorted out by testing the reaction kinetics and steady-state currents towards dopamine by cyclic voltammetry. Each electrode was then tested by cyclic voltammetry (0 to 0.8 V vs Ag/AgCl, 100 mV/s) in PBS (pH 7.4). Only those electrochemical nanosensors showing similar stable steady-state currents in Figure S1A were used for further electrochemical experiments.

Preparation of nanosensor 2: Platinization of nanosensor 1 to fabricate nanosensor 2 was previously described.[15, 54] Briefly, platinizing solution was prepared by PBS added with 1.5% H_2PtCl_6. The CNEs were scanned from 0 to −500 mV vs Ag/AgCl reference electrode at a scan rate of 2 mV/s for 1 cycle using a CHI electrochemical analyzer (CH Instruments, Inc., Austin, TX, USA). The electrodes were then tested by cyclic voltammetry (-0.1 to 0.8 V vs Ag/AgCl, 100 mV/s) in a solution of 1 mM H_2O_2 in PBS (pH 7.4). Only those electrochemical nanosensors showing similar stable steady-

state currents in Figure S1D were used for further electrochemical experiments. The fabrication success rate of functional nanosensors is approximately 80%, based on routine electrode preparation and electrochemical screening.

1.8. Electrochemical measurements

Vesicle impact electrochemical cytometry (VIEC) and stress granule impact electrochemical cytometry (SGIEC) measurements: Nanosensor 1 or 2 combined with an Ag/AgCl reference electrode (Scanbur, Sweden) are immersed in a suspension of SGs, vesicles or freshly mixed SGs and vesicles to perform VIEC measurements at a chosen potential applied by an Axopatch 200B potentiostat (Molecular Devices, Sunnyvale, CA). The output was filtered at 2.1 kHz and digitized at 5 kHz (Axoscope 10.4 software, Axon Instruments Inc., Sunnyvale, CA, USA). All VIEC experiments were carried out inside a Faraday cage.

Intracellular vesicle impact electrochemical cytometry (IVIEC) and intracellular stress granule impact electrochemical cytometry (ISGIEC)measurements: PC12 cells were stressed with 100 μm arsenite for 1 h or 100 μm cisplatin for 24 h to trigger acute or chronic SG assembly. Before performing IVIEC or ISGIEC measurement, the medium was removed, and cells were rinsed three times with the isotonic saline solution. The cells were kept at 37 °C in isotonic solution during the whole experimental process. All experiments were observed under an inverted microscope (IX81, Olympus) with 10x and 40x objectives. Nanosensor 1 or 2 was first placed on the top of a PC12 cell, and the tip was slowly pressed through the membrane of the cell while the current was recorded. Here, a potential of +700 mV or +300 mV vs. Ag/AgCl was applied on Nanosensor 1 or 2 to measure vesicles or SGs intracellularly, respectively. The data sampling rate was 10 kHz.

1.9. Data analysis

Amperometry traces were converted into txt by Matlab (The MathWorks, Inc.) and then processed by Igor Pro 6.22 (Wavemetrics, Lake Oswego, OR). The filter for the current was 1 kHz (binomial sm). The traces were carefully inspected after peak detection and false positives were manually rejected. The number of molecules from single vesicles, $t_{1/2}$, I_{max}, t_{rise}, and t_{fall} were pooled, and the median of the data was calculated for each experimental condition. To compare between different conditions, means of medians of the above parameters were calculated, groups were statistically analyzed with the Mann–Whitney rank sum test (unpaired and two-tailed) using Prism 7 (GraphPad, La Jolla, CA) (1 ***, $p < 0.001$; **, $p < 0.01$; *, $p < 0.05$). For fluorescence images, a negative control, which was only stained with secondary antibody, was included to determine the background signal to subtract from all the acquired images. The signal for G3BP1 was normalized to the nuclear signal, by dividing the number of G3BP1 total pixels by the total nuclear pixels to compensate for the varying number of cells in the image.

2. Supporting Figures and Tables

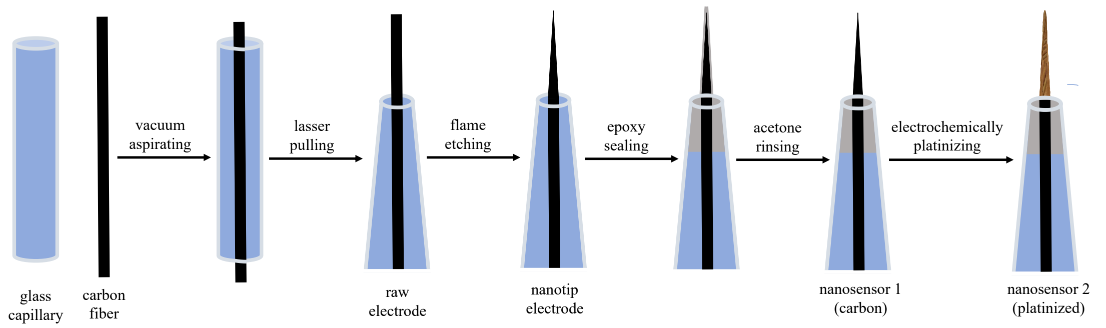

Figure S1. Schematic illustration of the fabrication of nanosensor 1 from carbon fiber and nanosensor 2 by platinization of nanosensor 1.

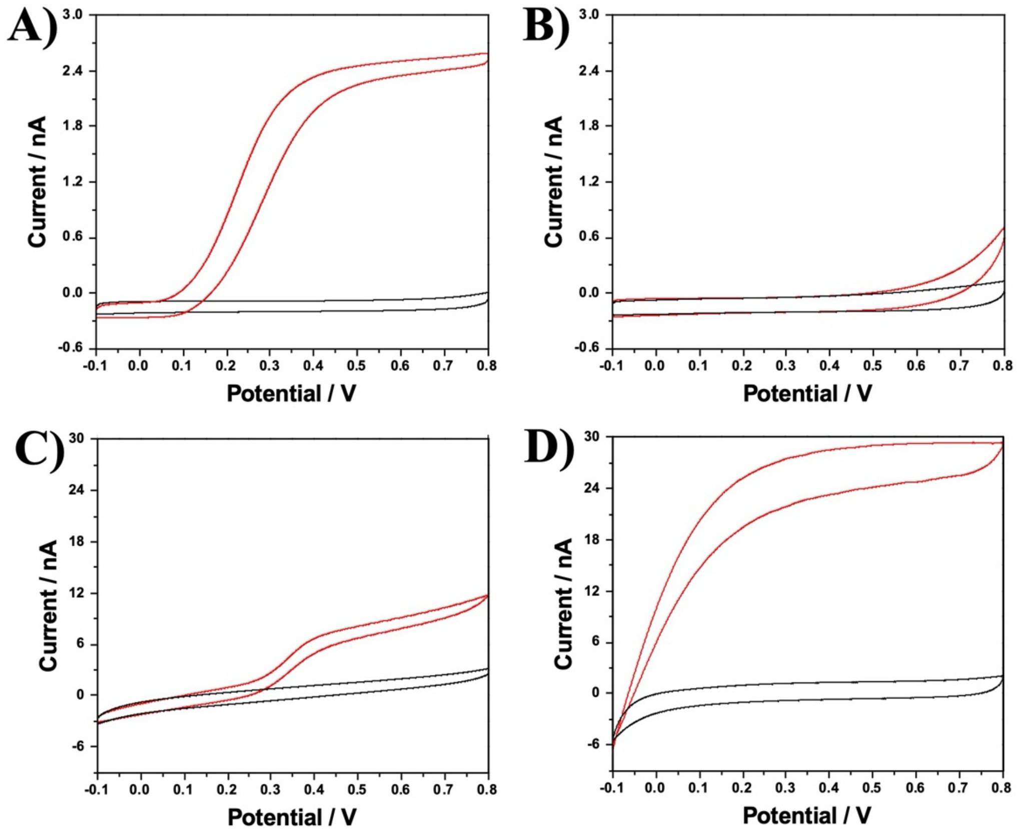

Figure S2. Representative cyclic voltammograms of nanosensor electrodes recorded in PBS before and after dopamine or H_2O_2. A–B) nanosensor 1 in PBS upon addition of A) 100 µM dopamine and B) 10 µM H_2O_2. C–D) Nanosensor 2 in PBS upon addition of C) 100 µM dopamine and D) 10 µM H_2O_2. Scan rate: 0.1 V/s. All potentials are reported versus Ag/AgCl.

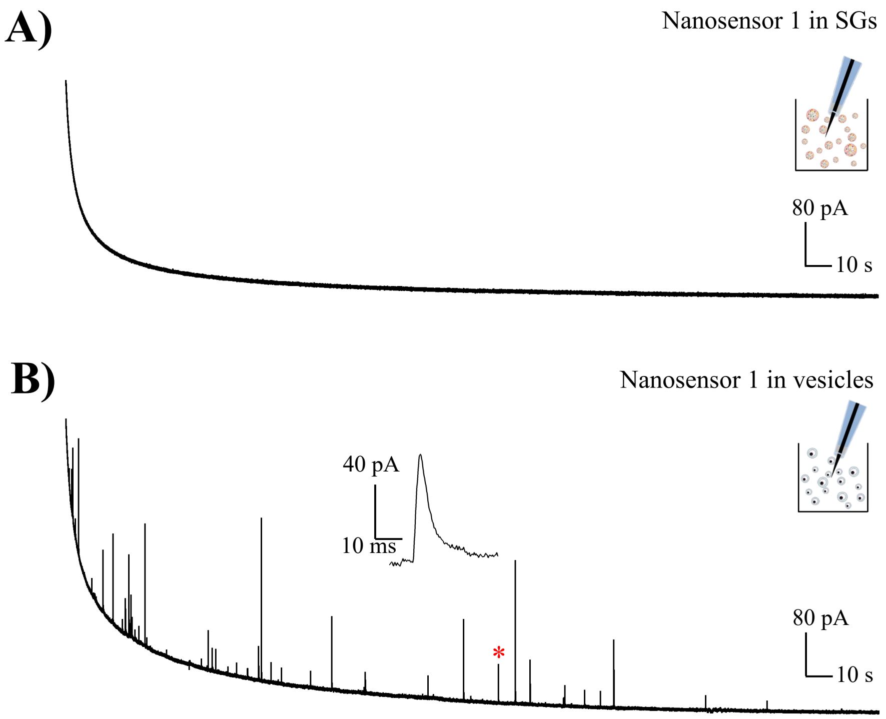

Figure S3. Representative amperometric traces recorded with nanosensor 1 at +700 mV (vs. Ag/AgCl) in suspensions of A) SGs and B) chromaffin vesicles. Inset: enlarged view of the spike labeled with the red asterisk.

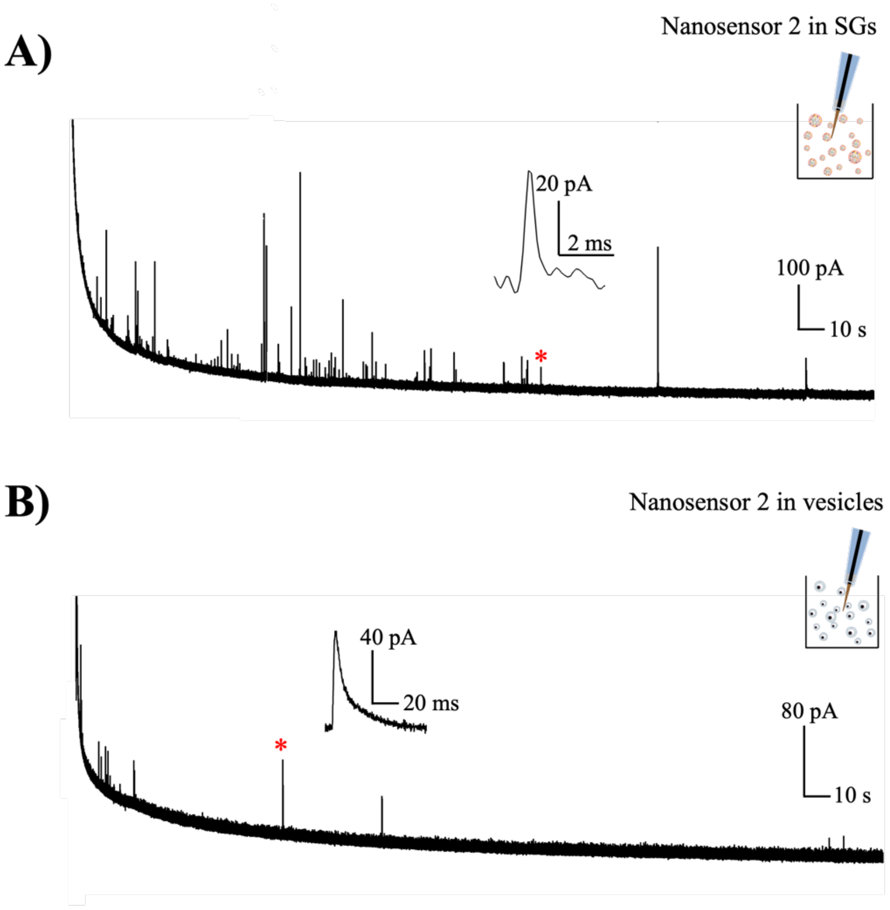

Figure S4. Representative amperometric traces recorded with nanosensor 2 at +400 mV (vs. Ag/AgCl) in suspensions of A) SGs and B) chromaffin vesicles. Inset: enlarged view of the spike labeled with the red asterisk.

Table S1. Statistical results of spike counts recorded from two-nanosensor in chromaffin vesicle suspensions at different applied potentials. Data were collected from 6 independent vesicle isolations.

Events Number	Number of nanosensor 1	Number of nanosensor 2		
	+700 mV	+400 mV	+350 mV	+300 mV
**None**	0	7	14	19
**1~5**	0	4	3	0
**5~10**	0	6	2	0
**>10**	19	2	0	0
**In Total**	19	19	19	19

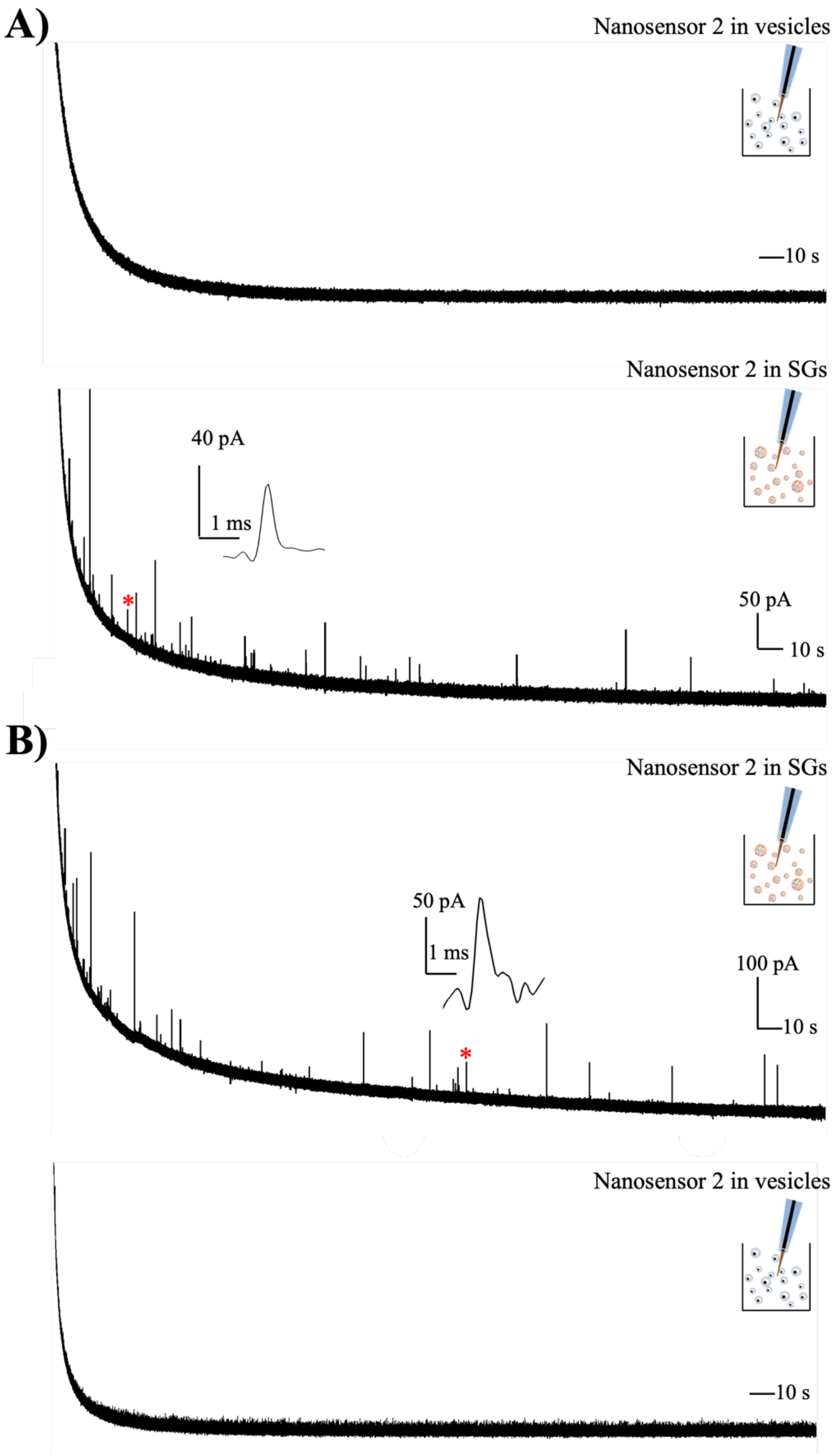

Figure S5. A) Representative amperometric traces recorded with the same nanosensor 2 at +300 mV (vs. Ag/AgCl) sequentially in suspensions of chromaffin vesicles (top) and SGs (bottom). B) Representative traces from a different nanosensor 2 exposed sequentially to SGs (top) and vesicles (bottom) under identical conditions. Inset: enlarged view of the spike labeled with the red asterisk.

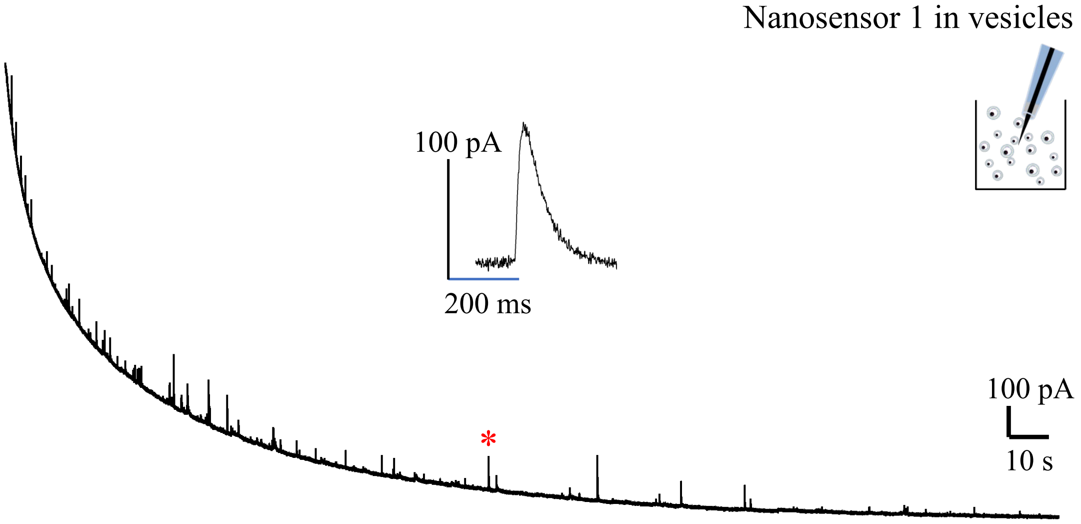

Figure S6. Representative amperometric trace recorded with nanosensor 1 at +300 mV (vs. Ag/AgCl) in a suspension of chromaffin vesicles. Inset: enlarged view of the spike labeled with the red asterisk.

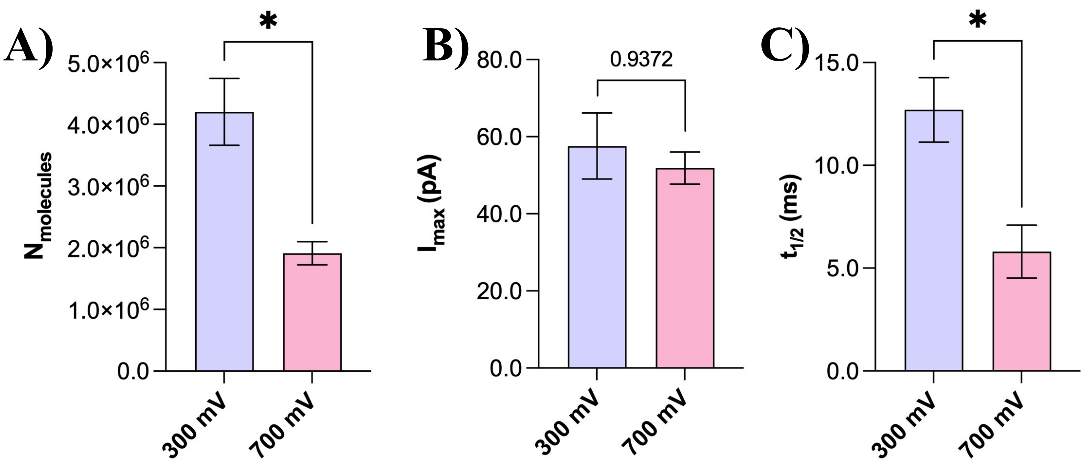

Figure S7. Bar graphs showing statistical medians of vesicle parameters obtained by nanosensor 1 in chromaffin vesicle suspensions at +300 mV and +700 mV (vs. Ag/AgCl): A) $N_{molecule}$, B) I_{max}, and C) $t_{1/2}$ (n=6 nanosensors per condition). Data are presented as mean ± SEM. Error bars represent SEM. Statistical significance was assessed using the Mann-§Whitney U test (two-tailed) for unpaired, non-normally distributed data: *$p < 0.05$.

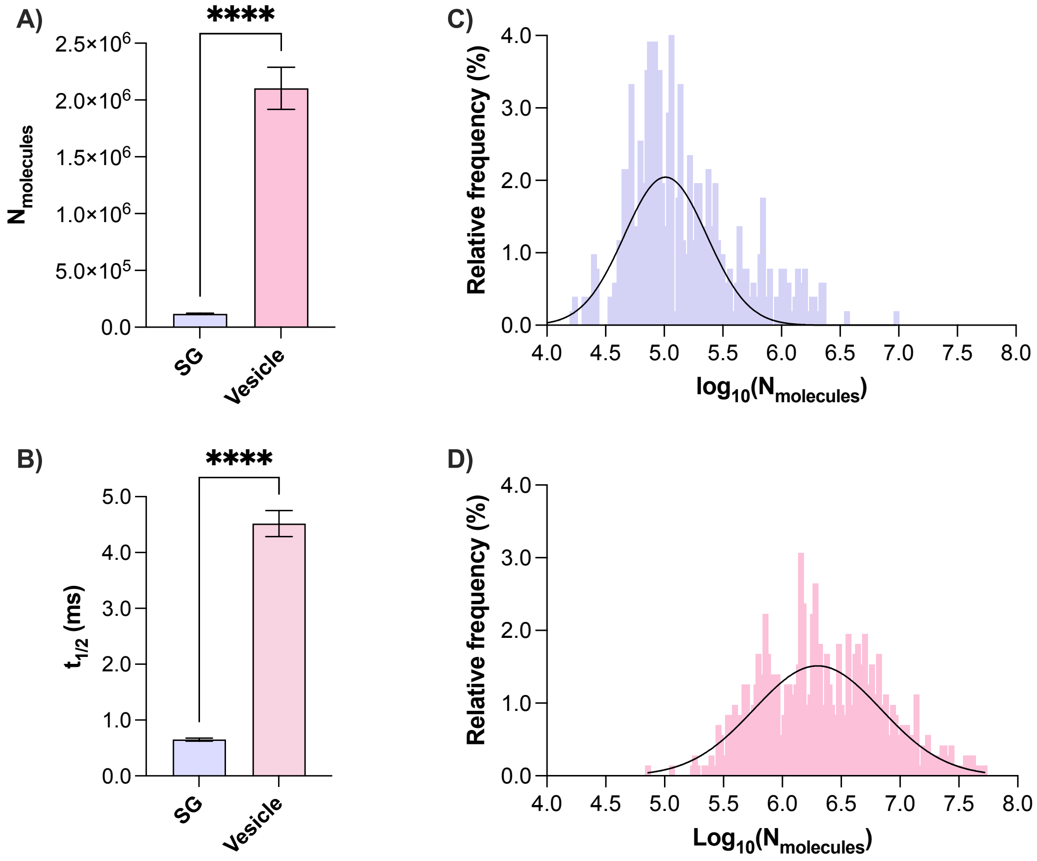

Figure S8. Bar graphs showing statistical medians of vesicle parameters obtained by nanosensor 1 at +700 mV (vs. Ag/AgCl) in chromaffin vesicle suspension and nanosensor 2 at +300 mV (vs. Ag/AgCl) in SG suspension separately: A) $N_{molecules}$ and B) $t_{1/2}$ (n=17 nanosensors per condition). Distribution of $log_{10}(N_{molecules})$ for C) SGs (n = 514 SGs) and D) vesicles (n = 718 vesicles), fitted using Gaussian models. Data are presented as mean ± SEM. Statistical significance was assessed using the Mann-Whitney U test (two-tailed) for unpaired, non-normally distributed data: ****$p < 0.0001$.

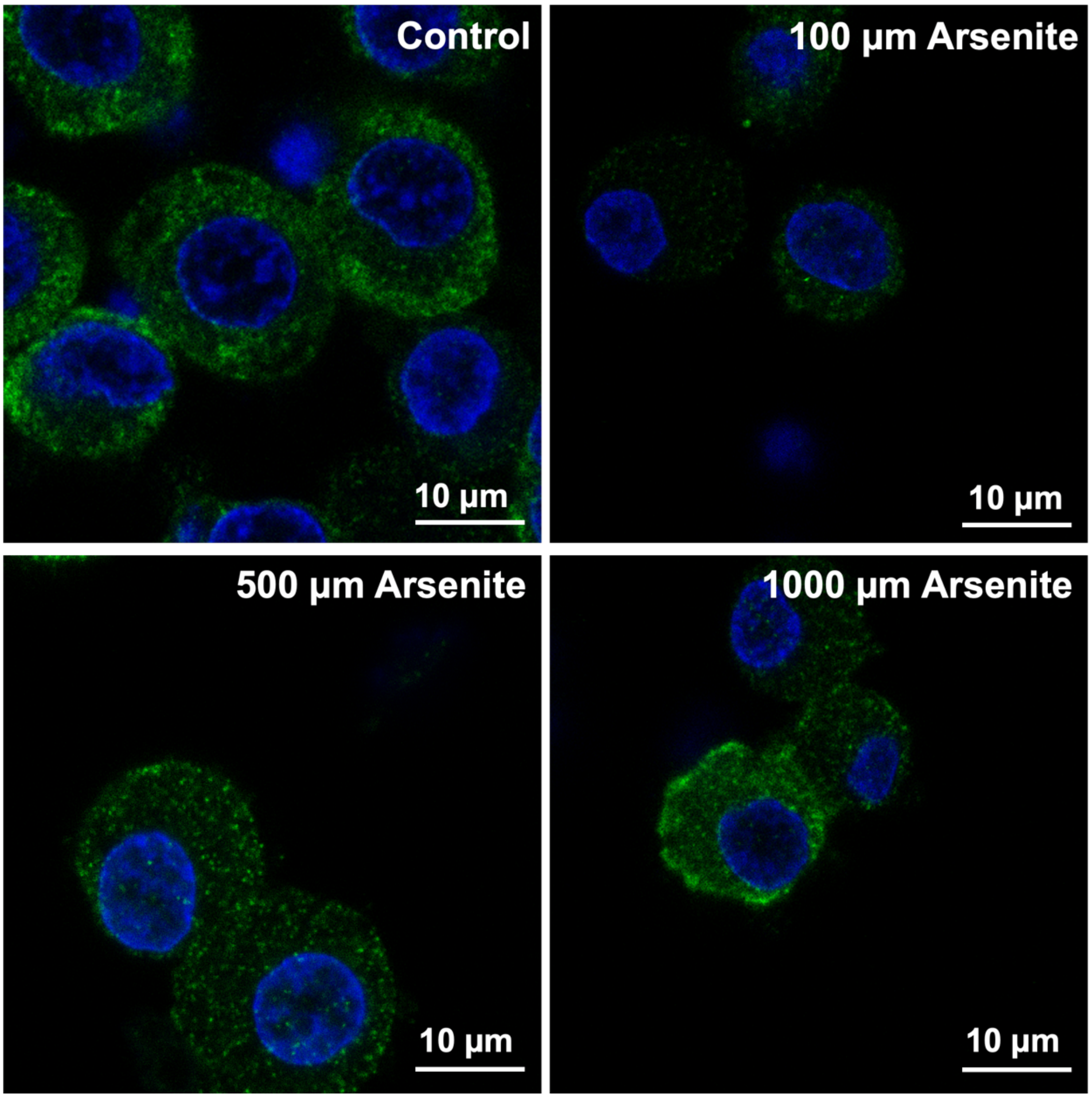

Figure S9. Representative fluorescence microscopy images of chromaffin cells treated with different concentrations of arsenite. After treatment, cells were fixed and SGs were visualized by immunofluorescence staining of G3BP1 (green, a canonical SG marker).

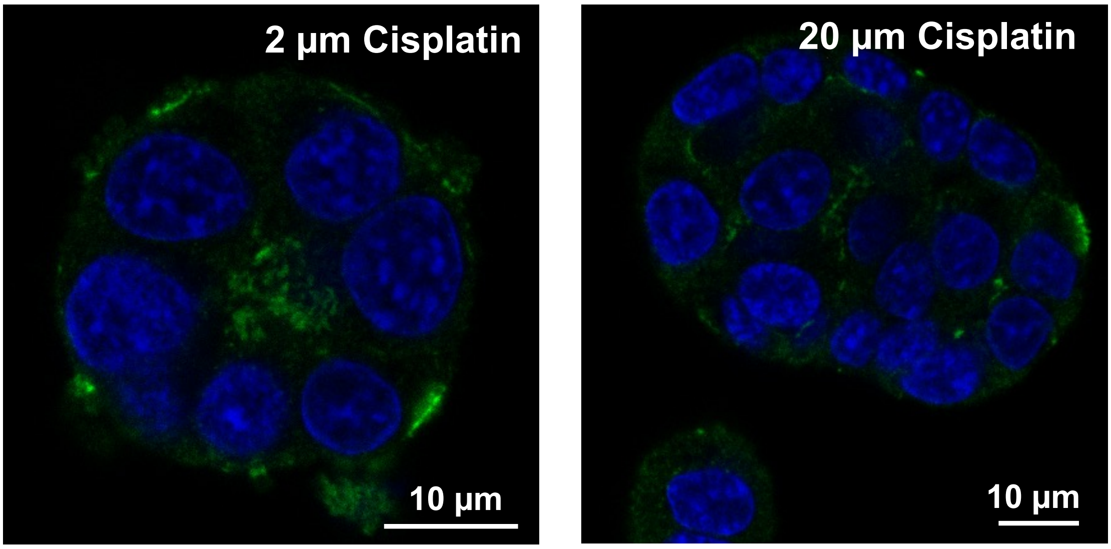

Figure S10. Representative fluorescence microscopy images of PC12 cells treated with 2 μM or 20 μM cisplatin for 24 h. After treatment, cells were fixed and SGs were visualized by immunofluorescence staining of G3BP1 (green, a canonical SG marker).

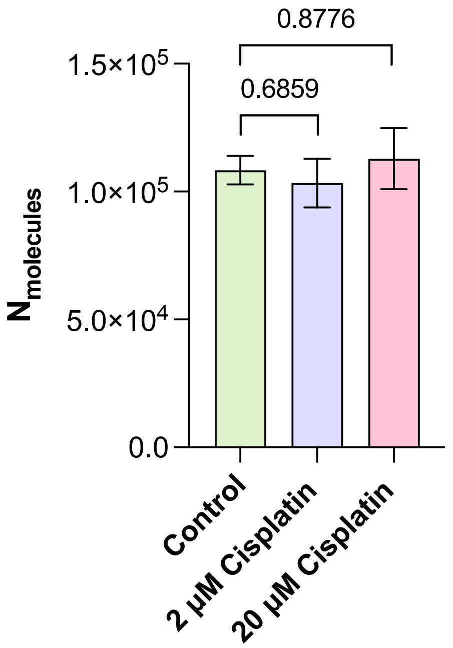

Figure S11. Bar graphs showing the statistical medians of catecholamine content per vesicle obtained from IVIEC measurements in PC12 cells treated with different concentrations of cisplatin (n=7 cells per condiction). Data are presented as mean ± SEM. Error bars represent SEM. Statistical significance was assessed using the Mann-Whitney U test (two-tailed) for unpaired, non-normally distributed data.

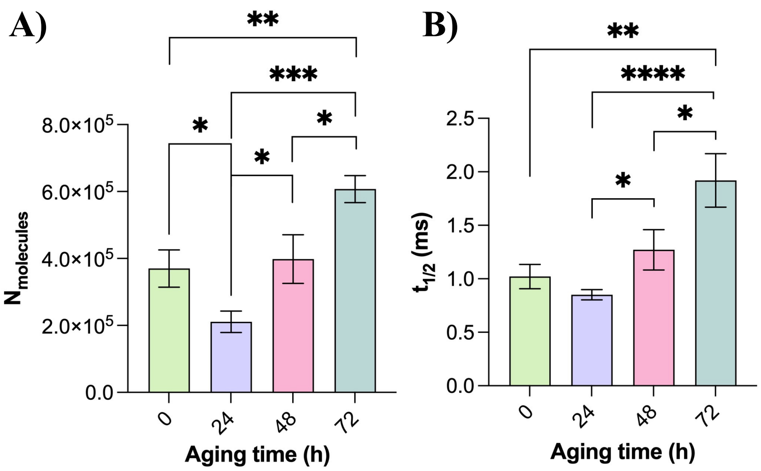

Figure S12. Comparison of H_2O_2 molecule numbers detected in SGs aged at 4 °C for 72 h. Conditions: 0, 24 h, 48 h, 72 h (n=8 nanosensors 2 per condiction). Data are presented as mean ± SEM. Error bars represent SEM. Statistical significance was assessed using the Mann-Whitney U test (two-tailed) for unpaired, non-normally distributed data: *p < 0.05, **p < 0.01, ***p < 0.001, ****p < 0.0001.

3. References

[14] K. Hu, E. Relton, N. Locker, N. T. N. Phan, A. G. Ewing, Electrochemical Measurements Reveal Reactive Oxygen Species in Stress Granules**. *Angew. Chem. Int. Ed*. **2021**, *60*, 15302-15306.

[15] H. Gu, C. Gu, A. Du Toit, W. Yu, M. W. Chen, H. L. Struckman, J. R. Silva, Y. Dai, A. G. Ewing, Single-Entity Resolution Single-Cell Nanosensor Reveals Reactive Oxygen Species at Stress Granules Are Formed by Interfacial Redox Chemistry. *J. Am. Chem. Soc*. **2025**, *147*, 27020-27029.

[16] H. Gu, C. Gu, N. Locker, A. G. Ewing, Amperometry and Electron Microscopy show Stress Granules Induce Homotypic Fusion of Catecholamine Vesicles. *Angew. Chem. Int. Ed*. **2024**, *63*, e202400422.

[17] X. Li, S. Majdi, J. Dunevall, H. Fathali, A. G. Ewing, Quantitative Measurement of Transmitters in Individual Vesicles in the Cytoplasm of Single Cells with Nanotip Electrodes. *Angew. Chem. Int. Ed*. **2015**, *54*, 11978-11982.

[18] J. Dunevall, H. Fathali, N. Najafinobar, J. Lovric, J. Wigström, A.-S. Cans, A. G. Ewing, Characterizing the Catecholamine Content of Single Mammalian Vesicles by Collision–Adsorption Events at an Electrode. *J. Am. Chem. Soc*. **2015**, *137*, 4344-4346.

[54] K. T. Kawagoe, J. A. Jankowski, R. M. Wightman, Etched Carbon-Fiber Electrodes as Amperometric Detectors of Catecholamine Secretion from Isolated Biological Cells. *Anal. Chem*. **1991**, *63*, 1589-1594.
